# Prevalence and association of self-reported anxiety, pain, and oral parafunctional habits with temporomandibular disorders in Japanese children and adolescents: a cross-sectional survey

**DOI:** 10.1186/1472-6831-15-8

**Published:** 2015-01-21

**Authors:** Hiroyuki Karibe, Kisaki Shimazu, Ayuko Okamoto, Tomomi Kawakami, Yuichi Kato, Sachie Warita-Naoi

**Affiliations:** Department of Pediatric Dentistry, School of Life Dentistry, Nippon Dental University, 1-9-20 Fujimi, Chiyoda-ku, Tokyo, 102-8159 Japan

**Keywords:** Temporomandibular disorders, Adolescents, Orofacial pain, Anxiety, Clenching

## Abstract

**Background:**

Associations between temporomandibular disorder (TMD) and psychological variables, pain conditions, and daily activities have been reported more commonly in middle-aged individuals than in children. However, to determine factor-specific preventive programs for TMD, it is important to evaluate the associations between multiple factors and TMD symptoms during childhood. The aim of this study was to assess the relationship between TMD symptoms and other orofacial pain conditions, daily activities, and trait anxiety in a population-based cross-sectional survey of Japanese children and adolescents.

**Methods:**

A total of 1,415 subjects (11–15 years old) self-reported their TMD symptoms, headache, neck pain, and toothache, and completed questionnaire scales that assessed 15 daily activities. Trait anxiety was assessed using the State Trait Anxiety Inventory for Children-Trait (STAIC-T) scale. Subjects were dichotomized into a TMD group or control group, based on whether they reported at least 1 TMD symptom: the TMD group (≥1 TMD symptom, n = 182) and the control group (no TMD symptoms, n = 1,233). Data were analyzed using the chi-square test and multivariate logistic regression analysis.

**Results:**

The prevalence rates for headache and neck pain were significantly higher in the TMD group than in the control group (44.0% vs. 24.7% and 54.4% vs. 30.0%, respectively; both *P* < 0.001). The odds ratios for TMD symptoms in subjects with neck pain and frequent diurnal clenching were 2.08 (*P* < 0.001) and 3.69 (*P* = 0.011), respectively. Moreover, high STAIC-T scores were weakly associated with TMD symptoms.

**Conclusions:**

In this young Japanese population, TMD symptoms were associated with other orofacial pain conditions, particularly neck pain, although they were only weakly associated with trait anxiety. Diurnal clenching was strongly associated with TMD symptoms. Health professionals should carefully consider these factors when developing appropriate management strategies for TMD in children and adolescents.

## Background

Temporomandibular disorders (TMDs) include several musculoskeletal and neuromuscular conditions that involve the masticatory muscles, the temporomandibular joint (TMJ), and the associated structures [1]. The signs and symptoms of TMD are frequently observed in children and adolescents [2, 3], and most adult patients with TMDs report that these symptoms developed during their adolescence. In addition, these signs and symptoms can be prolonged by various anatomical, pathophysiological, and psychosocial factors [4].

Prolonged masticatory muscle pain is likely to become a chronic condition, and continuous pain may eventually produce chronic centrally mediated myalgia [1]. Unfortunately, patient with chronic TMD frequently develop symptoms of other painful face/head conditions (or in other parts of the body), and can also develop psychological comorbidities [5, 6]. For example, one previous study of young patients with TMD revealed an association between myofascial pain and headache [7]. Furthermore, postural imbalance and parafunctional habits have been suggested to induce TMD symptoms in adults [8]. Although population-based studies have previously examined the associations between TMD and psychological variables, pain conditions, and oral parafunctional habits [9, 10], the symptoms of TMD have been more commonly reported in middle-aged individuals, rather than in children [11].

Our recent study [12] reported that 16- to 18-year-old adolescents with TMDs experience higher pain intensity in the orofacial region and greater difficulty in their activities of daily living than 13- to 15-year-old adolescents with TMDs. In addition, the symptoms of TMDs in the 16- to 18-year-old adolescents were very similar to those experienced by adults. Furthermore, to develop appropriate factor-specific preventive programs for TMD in children and early adolescents, it is important to carefully evaluate the associations between multiple factors and TMD symptoms during childhood. However, the prevalence of TMD in children who are ≤10 years old is relatively low [13], and these younger children may be unable to read the research questionnaire [14]. Therefore, we aimed to investigate the prevalence of TMD symptoms in Japanese children and adolescents who were 11–15 years old, and to assess the relationships between TMD symptoms and other orofacial pain conditions, daily activities, and trait anxiety in a population-based, cross-sectional survey. In this study, we tested the hypothesis that, even in a young population, TMD symptoms are associated with head, neck, and tooth pain; trait anxiety; postural imbalance; and parafunctional habits.

## Methods

### Study subjects

This study included Japanese children and adolescents (11–15 years old) from a regional survey of 1,678 students who attended 3 elementary schools and 3 junior high schools in Suginami, Tokyo. Among the 43 elementary and 23 junior high schools that we approached, these 6 schools were selected because their administration consented to participate in this study. No schools with mentally challenged or learning-disabled students were included. Among the 1,678 students who were invited and participated in this study, data from 263 students who missed 1 or more questionnaire items were excluded. Therefore, the final sample was comprised of data from 1,415 students. This sample was representative of the 16,917 students (11–15 years old) who attended elementary schools and junior high schools in Suginami during 2011. All data were collected at the students’ annual dental check-ups, which were conducted between October and November 2011, at all 6 schools.

The study protocol was reviewed and approved by the Ethics Committee at the Nippon Dental University School of Life Dentistry (NDU-T2011-21), and the local education authority, and conformed to the guidelines of the Declaration of Helsinki. The students and their parents provided their informed consent prior to participation.

### Measurements

The primary outcome was self-reported TMD symptoms, as determined using a questionnaire that was based on the “Research Diagnostic Criteria for Temporomandibular Disorders” [15]. This questionnaire consisted of 6 questions: 3 questions regarding TMD symptoms (i.e., jaw pain, TMJ sounds, and limited jaw opening) during the previous 3 months, and 3 questions regarding related pain conditions (i.e., headache, neck pain, and toothache) during the previous 3 months. As the questionnaire was originally developed in English, and Japanese was the common language for the study subjects, the questionnaire was translated into plain Japanese by the authors. To confirm that the English and Japanese questionnaires had the same contents, the initial translation into Japanese was re-translated into English by bilingual faculty members, and the contents of the original English and re-translated English versions were compared to ensure consistency in the questions. All versions were also analyzed and compared by the authors, a final version was obtained, and the equivalence of its language was assessed using the test-retest method. According to this method, 16 volunteers received 2 versions (English and Japanese) of the questionnaire, and they were instructed to complete the first version (English or Japanese) on the same day they received the questionnaires, and to complete the second version (Japanese or English) on the next day, without referring to the previous questionnaire. The kappa coefficient was used to evaluate the language equivalency, and 5 of the 6 questions provided an average kappa value of 0.75. Among these questions, 1 question provided a kappa value of 1, while the other 4 questions provided kappa values of 0.53 to 0.87. For the remaining question, it was not possible to calculate the kappa coefficient, as all volunteers provided identical responses to this question for both versions. These results indicated a good equivalency between the 2 versions of the questionnaire. (A copy of the Japanese questionnaire is available to interested researchers from the corresponding author.) Subjects were dichotomized into a TMD group (n = 182) or control group (n = 1,233), based on whether they reported at least 1 TMD symptom.

To assess ordinary daily life, a questionnaire was developed by the authors based on the contents of a patient education and self-management program regarding the musculoskeletal system [1, 16]. This questionnaire consisted of 15 statements that assessed the subjects’ normal daily routines regarding eating (gum chewing and eating hard foods), posture (sitting at a desk for >2 h, playing video games for >1 h, head-forward posture, and resting the chin on a hand), oral habits (diurnal clenching and nocturnal tooth grinding), sleeping habits (sleeping in a prone position, using a hard pillow, and using a high pillow), and extracurricular activities (voice training, playing a musical instrument using the jaw/mouth, exercising >3 times per week, and studying late at night). Each statement was rated on a 5-point scale (1: never; 2: a little; 3: somewhat; 4: often; 5: always). To determine the test-retest reliability of the questionnaire, we calculated the intraclass correlation coefficient (ICC), in which subjects and occasions were considered random factors [17]. The average ICC for all questionnaire items was 0.84. According to the common scale for ICC, the test-retest reliability was excellent for 13 questionnaire items (ICCs of >0.75) and fair-to-good for 2 items (ICCs of 0.4 and 0.75); these results indicate that the questionnaire was reliable.

To assess trait anxiety, the participants completed the relevant scales from the State-Trait Anxiety Inventory for Children-Trait (STAIC-T) questionnaire [18], which are comprised of self-reported scales for measuring trait anxiety. Trait anxiety is defined as an individual’s tendency to react in an anxious way, regardless of the situation [19]. The STAIC-T scale consists of 20 statements, and the participants responded to the STAIC-T statements using a 3-point rating scale (1–3). Thus, scores for the STAIC-T subscale range from 20 to 60, with higher scores indicating greater anxiety. The psychometric test used in this study was the Japanese version of STAIC-T, and its validity has previously been verified among children who are ≥10 years old [14].

### Statistical analyses

Differences in age and STAIC-T scores were compared between the 2 groups using Student’s *t*-test. The chi-square test was used to analyze group differences regarding sex and the incidence headache, neck pain, toothache, and 15 daily activities. A *P*-value of <0.05 was considered statistically significant. For sex, headache, neck pain, toothache, and the 15 daily activities, logistic regression was used to test the univariate activity-symptom relationships and multivariate association with self-reported TMD symptoms. To test for differences among the daily activity categories, daily activity was modeled with indicator variables, using a no activity category (i.e., “Never”) as the reference. Variables were considered for inclusion in the multivariate models if their univariate *P*-value was <0.10. All analyses were performed using a statistical software package (IBM SPSS Statistics, version 21, IBM Japan, Tokyo, Japan).

## Results

TMD symptoms were reported by 12.9% of the participants who were included in our analysis (182/1,415). Table 1 lists the mean age, trait anxiety score, and relevant symptoms for the 2 groups. Subjects in the TMD group were slightly older and reported higher trait anxiety scores than those in the control group (both, *P* < 0.001). In addition, there were significantly more girls in the TMD group than in the control group (*P* = 0.014). The prevalence rates for headache, neck pain, and toothache were significantly higher in the TMD group than those in the control group (*P* < 0.001, *P* < 0.001, and *P* = 0.009, respectively). Univariate logistic regression analysis revealed that female sex (odds ratio [OR]: 1.48; 95% confidence interval [CI]: 1.08–2.02; *P* = 0.014), headache (OR: 2.40; 95% CI: 1.74–3.30; *P* < 0.001), neck pain (OR: 2.78; 95% CI: 2.03–3.82; *P* < 0.001), and toothache (OR: 1.80; 95% CI: 1.15–2.82; *P* = 0.010) might influence the TMD symptoms.

**Table 1:** **Sample characteristics and prevalence of other pain conditions according to temporomandibular disorder symptom status**

	TMD group (n = 182)	Control group (n = 1,233)	***P***-value*
Age (years)	13.6 ± 1.2	13.2 ± 1.4	<0.001
STAIC-T score	38.9 ± 9.3	35.2 ± 7.8	<0.001
Sex			
Male	82 (45.1)	676 (54.8)	0.014
Female	100 (54.9)	557 (45.2)	
Headache			
No	102 (56.0)	929 (75.3)	<0.001
Yes	80 (44.0)	304 (24.7)	
Neck pain			
No	83 (45.6)	863 (70.0)	<0.001
Yes	99 (54.4)	370 (30.0)	
Toothache			
No	154 (84.6)	1120 (90.8)	0.009
Yes	28 (15.4)	113 (9.2)	

*t-test or chi-square test, TMD: temporomandibular disorder, STAIC-T: State-Trait Anxiety Inventory for Children–Trait.

The frequency distributions for head-forward posture, resting the chin on a hand, diurnal clenching, nocturnal tooth grinding, and sleeping in a prone position were significantly different when the TMD and control groups were compared (chi-square test: *P* = 0.001, *P* < 0.001, *P* < 0.001, *P =* 0.008, and *P =* 0.015, respectively). Table 2 lists the frequency distribution of head-forward posture, diurnal clenching, and nocturnal tooth grinding, as well as the univariate OR for the TMD symptoms. According to the univariate logistic regression analysis, gum chewing (always) (OR: 2.01; 95% CI: 0.93–4.35; *P* = 0.076), eating hard foods (always) (OR: 2.39; 95% CI: 1.06–5.38; *P* = 0.036), resting the chin on a hand (always) (OR: 4.22; 95% CI: 2.16–8.25; *P* < 0.001), sleeping in a prone position (always) (OR: 2.11; 95% CI: 1.24–3.60; *P* = 0.006), and voice training (always) (OR: 1.98; 95% CI: 1.06–3.71; *P* = 0.032) might influence the TMD symptoms.

**Table 2:** **Odds ratios for temporomandibular disorder symptoms according to the frequency of daily activities**

	TMD group n (%)	Control group n (%)	OR (univariate)	95% CI	***P***-value
Head-forward posture					
Never	32 (17.6)	270 (21.9)	reference		
A little	24 (13.2)	231 (18.7)	0.88	0.50–1.53	0.64
Somewhat	46 (25.3)	309 (25.1)	1.26	0.78–2.03	0.35
Often	27 (14.8)	216 (17.5)	1.06	0.61–1.81	0.85
Always	53 (29.1)	207 (16.8)	2.16	1.34–3.47	0.001
Diurnal clenching					
Never	106 (58.2)	968 (78.5)	reference		
A little	32 (17.6)	157 (12.7)	1.86	1.21–2.86	0.005
Somewhat	29 (15.9)	76 (6.2)	3.49	2.17–5.59	<0.001
Often	8 (4.4)	20 (1.6)	3.65	1.57–8.50	0.003
Always	7 (3.8)	12 (1.0)	5.33	2.05–13.82	0.001
Nocturnal tooth grinding					
Never	128 (70.3)	992 (80.5)	reference		
A little	27 (14.8)	124 (10.1)	1.69	1.07–2.66	0.024
Somewhat	13 (7.2)	77 (6.2)	1.31	0.71–2.42	0.39
Often	8 (4.4)	24 (1.9)	2.58	1.14–5.87	0.023
Always	6 (3.3)	16 (1.3)	2.91	1.12–7.56	0.029

TMD: temporomandibular disorder, OR: odds ratio, CI: confidence interval.

Age, STAIC-T score, sex, headache, neck pain, toothache, and the 8 daily activities (head-forward posture, diurnal clenching, nocturnal tooth grinding, gum chewing, eating hard foods, resting the chin on a hand, sleeping in a prone position, and voice training) were included in the multivariate model. Among these variables 5 factors significantly influenced the occurrence of TMD symptoms (Table 3), including age (OR: 1.24; 95% CI: 1.09–1.40), female sex (OR: 1.40; 95% CI: 1.01–1.94), STAIC-T score (OR: 1.03; 95% CI: 1.01–1.05), reported neck pain (OR: 2.08; 95% CI: 1.49–2.90), and habitual diurnal clenching (always) (OR: 3.69; 95% CI: 1.34–10.13).

**Table 3:** **Multiple logistic regression analysis of risk factors for self-reported temporomandibular disorder symptoms**

	***B***	Wald	OR	95% CI	***P***-value
Age	0.22	11.33	1.24	1.09–1.40	0.001
Sex (female)	0.33	3.97	1.40	1.01–1.94	0.046
STAIC-T score	0.03	7.37	1.03	1.01–1.05	0.007
Neck pain (yes)	0.73	18.53	2.08	1.49–2.90	<0.001
Diurnal clenching (always)	1.30	6.39	3.69	1.34–10.13	0.011

*B*: regression coefficient, OR: odds ratio, CI: confidence interval, STAIC-T: State-Trait Anxiety Inventory for Children-Trait.

## Discussion

In this population-based cross-sectional study, we assessed the relationships between TMD symptoms and other orofacial pain conditions, daily activities, and trait anxiety in Japanese children and adolescents. Our hypotheses were partially confirmed by the findings: even in this relatively young population, subjects with TMD symptoms had a higher prevalence of other pain conditions and parafunctional habits, compared to subjects without TMD symptoms.

The prevalence of TMD symptoms in young subjects varies widely, due to inter-subject variation and the use of different analysis methods. For example, several studies have reported that TMD symptoms were observed in 16–33% of the non-patient adolescent population [20–22]. In addition, a study of 3,292 Japanese students (11–15 years old) reported that TMD symptoms were observed in nearly 12% of the subjects [13]. This result is consistent with the results of the present study, which indicate that 12.9% of the subjects had at least 1 TMD symptom.

Epidemiological studies have reported that the prevalence of TMD symptoms declines with age [4, 11, 23], and several TMD symptoms have been shown to be age-dependent among adults who are 20–70 years old. For example, TMJ clicking was apparent in subjects who were 30–40 years old, and jaw/face pain was the most prevalent symptom in subjects who were 40–50 years old [11]. In the present study, all subjects were 11–15 years old, and while we did not specifically investigate the age-dependent symptoms, the prevalence of TMD symptoms did increase with age in our study population.

Regarding sex-related differences, previous studies have reported that TMD-related pain and other symptoms were more common in girls compared to in boys [3, 24]. This fact may be related to neuropsychological and physiological factors, as women appear to have a lower pain threshold and are more vulnerable to stress, compared to men [25]. Cross-sectional population-based studies have also revealed that pain and TMD symptoms increase with pubertal development in women [26, 27], and hormonal changes may also play an important role in the etiology of the disorder [28]. In addition, epidemiological studies in children and adolescents have reported that the female-sex OR for TMD-related symptoms ranged from 2.0 to 3.5 [29–31]. In the present study, the association between TMD symptoms and the female sex was relatively weak (OR = 1.4), which may reflect the differences in pubertal development among the study subjects.

Patients with myofascial TMDs have reported more severe symptoms of depression and anxiety, compared with those reported by normal, pain-free, individuals [32]. In addition, psychosocial factors, such as increased stress levels and emotional challenges, have been noted in adolescents with TMDs [33]. In the present study, subjects in the TMD group had significantly higher trait anxiety scores (38.9) than subjects in the control group (35.2). However, the mean STAIC-T scores for Japanese boys (35.5) and girls (37.5) [14] are very similar to the STAIC-T scores observed in the present study. Moreover, the trait anxiety OR (1.03, derived from the multivariate analysis) suggests that the association between TMD symptoms and trait anxiety is weak. Thus, the difference in the STAIC-T scores for the TMD and control groups appears to be clinically irrelevant. A previous study has also reported that subjective TMD and psychosomatic symptoms were weakly correlated in adolescents [34], which is consistent with the results of the present study. Therefore, the higher level of anxiety in children may not reflect these emotional challenges. However, the role of psychosomatic symptoms (including anxiety) in predisposing subjects to the development of TMD remains notable.

Adolescents with TMD are more likely to experience multiple bodily pains, compared to healthy subjects [33, 35]. In addition, headache, neck pain, and somatic complaints have been reported to be significantly associated with TMD pain in adolescents [36]. Furthermore, a recent study has reported that headaches were a potential risk factor for TMD in adolescents, and that the risk was particular high for individuals with chronic TMD [37]. In contrast, a longitudinal study of adolescents with or without headache reported that headache-related TMDs were not predictable [38], although headache appears to precede TMD pain in many adolescents [36]. In the present study, the prevalence of headache and neck pain was significantly higher in the TMD group than that in the control group, and the reported neck pain exhibited a high OR (2.08) for TMD symptoms. These results are consistent with those of previous studies in several aspects. For example, Svensson [6] stated that myofascial TMD pain and tension-type headache appear to share many of the same pathophysiological mechanisms, although it would be premature to consider them as identical entities. When headache and neck pain are reported by children and adolescents, a comprehensive examination is recommended to identify any TMD symptoms.

Numerous studies have suggested that TMD may be associated with a head-forward posture [39], and a study of myogenous TMD reported significant improvements in pain and jaw opening after the addition of postural exercise training [40]. Patients with TMD who exhibit a head-forward posture may experience symptom improvement after posture training or after being provided with self-management instructions [8]. In the present study, head-forward posture was significantly more frequent in the subjects with TMD than in the controls. However, multivariate analysis revealed that head-forward posture was not a significant risk factor for TMD symptoms, and the relationship between head-forward posture and TMD symptoms remains inconclusive [41].

Oral habits or parafunctional habits involve a variety of common activities (e.g., continuous gum chewing, nail biting, diurnal clenching, and nocturnal tooth grinding), and many children and adolescents perform them on a daily basis [42]. However, parafunctional habits may overload the masticatory system, and might also play an etiological role in the development of TMDs [43]. For example, diurnal clenching and sleep-related bruxism have been reported to be associated with TMDs in children and adolescents [10]. In addition, while several studies have reported a correlation between oral parafunctional habits and TMD symptoms in children and adolescents [44, 45], others have disputed these correlations [46, 47]. In the present study, habitual diurnal clenching significantly influenced the occurrence of TMD symptoms, although nocturnal tooth grinding did not. However, subjects may not be aware of nocturnal tooth grinding during sleeping, therefore behavior modification to control habitual diurnal clenching remains an important goal for treating and preventing TMD [48].

The present study has several limitations. First, we did not assess the duration or severity of TMD symptoms. Therefore, we could not evaluate the preventive effect of individualized self-care programs for TMD symptoms in this young population. The self-reporting of TMD symptoms might be another limitation, as the data we analyzed relied on participant recall and reporting. Thus, it is possible that there is a significant difference between the self-reported symptoms and clinically recorded signs. Therefore, a clinical evaluation of dental, muscular, and TMJ pathology would be preferable in future studies.

Although there are several limitations in this population-based, cross-sectional study, we observed a relatively high prevalence of TMD symptoms in 11- to 15-year-old Japanese children and adolescents. Based on our results, other pain conditions and parafunctional habits may exist that are related to TMD symptoms, even in this young population, and these factors should be managed swiftly. However, TMD symptoms frequently recur, and a follow-up study is needed to verify the prognosis for these TMD symptoms. In the future, we hope to conduct a longitudinal study to evaluate the effectiveness of strategies that have been developed to prevent TMD symptoms in children and adolescents.

## Conclusions

In children and adolescents, TMD symptoms were associated with various orofacial conditions, particularly neck pain. In contrast, TMD symptoms were only weakly associated with trait anxiety. Postural imbalance was not significantly associated with TMD symptoms, although habitual diurnal clenching greatly affected the TMD symptoms. Health professionals should carefully consider these factors when developing appropriate management strategies for TMD in children and adolescents.

## Abbreviations

TMD: Temporomandibular disorder
TMJ: Temporomandibular joint
ICC: Intraclass correlation coefficient
STAIC-T: State-Trait Anxiety Inventory for Children-Trait
OR: Odds ratio
CI: Confidence interval.
